# Imidazole-rich copper peptides as catalysts in xenobiotic degradation

**DOI:** 10.1371/journal.pone.0238147

**Published:** 2020-11-04

**Authors:** Sharifa Zaithun Begum, Nurul Shairah Mohd Nizam, Azira Muhamad, Mohd Izham Saiman, Karen Anne Crouse, Mohd Basyaruddin Abdul Rahman

**Affiliations:** 1 Integrated Chemical BioPhysics Research, Faculty of Science, Universiti Putra Malaysia, Serdang, Selangor, Malaysia; 2 Nanomolecular Laboratory, Faculty of Science, Universiti Putra Malaysia, Serdang, Selangor, Malaysia; 3 Structural & Biophysics Facility, Malaysia Genome Institute, National Institutes of Biotechnology Malaysia, Jalan Bangi, Selangor, Malaysia; 4 Department of Chemistry, Faculty of Science, Universiti Putra Malaysia, Serdang, Selangor, Malaysia; CIC bioGUNE, SPAIN

## Abstract

Laccases, oxidative copper-enzymes found in fungi and bacteria were used as the basis in the design of nona- and tetrapeptides. Laccases are known to be excellent catalysts for the degradation of phenolic xenobiotic waste. However, since solvent extraction of laccases is environmentally-unfriendly and yields obtained are low, they are less preferred compared to synthetic catalysts. The histidine rich peptides were designed based on the active site of laccase extracted from *Trametes versicolor* through RCSB Protein Data Bank, LOMETS and PyMol software. The peptides were synthesized using Fmoc-solid phase peptide synthesis (SPPS) with 30–40% yield. These peptides were purified and characterized using LC-MS (purities >75%), FTIR and NMR spectroscopy. Synthesized copper(II)-peptides were crystallized and then analyzed spectroscopically. Their structures were elucidated using 1D and 2D NMR. Standards (*o*,*m*,*p*-cresol, 2,4-dichlorophenol) catalysed using laccase from *Trametes versicolor* (0.66 U/mg) were screened under different temperatures and stirring rate conditions. After optimizing the degradation of the standards with the best reaction conditions reported herein, medications with phenolic and aromatic structures such as ibuprofen, paracetamol (acetaminophen), salbutamol, erythromycin and insulin were screened using laccase (positive control), apo-peptides and copper-peptides. Their activities evaluated using GC-MS, were compared with those of peptide and copper-peptide catalysts. The tetrapeptide was found to have the higher degradation activity towards salbutamol (96.8%) compared with laccase at 42.8%. Ibuprofen (35.1%), salbutamol (52.9%) and erythromycin (49.7%) were reported to have the highest degradation activities using Cu-tetrapeptide as catalyst when compared with the other medications. Consequently, *o*-cresol (84%) was oxidized by Tp-Cu while the apo-peptides failed to oxidize the cresols. Copper(II)-peptides were observed to have higher catalytic activity compared to their parent peptides and the enzyme laccase for xenobiotic degradation.

## Introduction

Pharmaceutical waste is an emerging environmental and health concern worldwide. Irresponsible drug disposal results in the leaching of active ingredients into the drinking water supply through landfills and/or into the atmosphere [1]. Traditional wastewater treatment involving filtration (physical), activated sludge process and aeration (chemical) and digestion by aerobic and anaerobic bacteria (biological) are not sufficient to completely remove the treatment-resistant, stable phenolic and cyclic pharmaceutically active compounds (PhACs) [2]. In addition, other classes of pharmaceutical compounds including analgesics, anti-depressants, anti-hypertensives, antibiotics and steroids have been detected in water rendering it unsafe for consumption [1]. In order to protect and sustain water sources for community supply and to reduce the amount and varieties of pharmaceutical effluents entering the drinking water reserves, alternative treatment methods to degrade or bioremediate the treatment-resistant cyclic and phenolic PhACs such as cresols, erythromycin, diphenylhyramine based cough syrup, *etc* have been studied in depth since the 20^th^ century [1].

In the past, various treatments have been applied to degrade prior to safe environmental disposal such as incineration of solid and aerosolized wastes, and flushing down liquid medicines [2]. However, due to incomplete degradation of PhACs using these techniques and insufficient education of patients in safe disposal of liquid wastes, the minute pharmaceutical effluents in these wastes still entered the drinking water system posing an environmental threat [3]. Huber [2] reported the successful treatment of drinking water supply by oxidation using 90–99% ozone (O_3_) at a concentration ≥ 2 mg/L [2]. Non-steroidal anti-inflammatory drugs (NSAIDs) such as ibuprofen, diclofenac and indomethacin are among the common pharmaceutical pollutants found in wastewater and in the atmosphere. Microbes have been used to degrade diclofenac anaerobically and mineralize ibuprofen aerobically before final treatment with activated sludge for their full removal [4]. Advanced oxidation processes (AOP) which include a combination of ozonation, photocatalysis, ultrasound oxidation and adsorption on activated carbon have also been employed to degrade recalcitrant compounds into CO_2_, H_2_O and inorganic ions [5, 6]. AOP is expensive and produces extremely reactive hydroxyl radicals, ·OH, that cause the depletion of ozone layer, making it environmentally unfriendly [7].

In order to address the issues mentioned above, oxidative enzymes were then employed as catalysts to degrade PhACs waste into biodegradable or inorganic waste. The choices of oxidative enzymes varied from human enzymes to gut microbiomes to oxidases and lignolytic fungal enzymes such as laccase [8]. Oxidases such as laccases have been reported to have the highest catalytic activity towards the degradation of recalcitrant aromatic PhACs compared to other oxidases such as horseradish peroxidase, lignin peroxidase and manganese peroxidase [9]. However, despite completely oxidizing the stable cyclic and aromatic PhACs, the peroxidases also emitted hydroxyl radicals as a side product of the oxidation reaction.

Laccase, (E.C.1.10.3.2, benzenediol: oxygen oxidoreductases) was reported in 2000 to be a successful catalyst in eliminating treatment-resistant, non biodegradable cyclic PhACs such as phenols to biodegradable ones, eventually forming CO_2_, H_2_O and inorganic ions through a polymerization process [9]. However, laccase requires the use of mediator ions such as 2,2`-azinobis (3-ethylbenzthiazoline-6-sulfonate) (ABTS) and N-hydroxybenzotriazole (HBT) or H_2_O_2_ to facilitate the oxidation and polymerization process. Without these mediators, laccase has low catalytic activity and is unable to completely oxidize or degrade the PhACs [10]. Laccases from different fungal sources behave differently as catalysts. The laccase reported to have the highest oxidation activity in xenobiotic degradation was extracted from the fungal source *Trametes versicolor* [3].

This discovery, together with the use of green chemistry where the use of mediator ions and vast amounts of solvents could be eliminated, sparked an interest in the synthesis of specific copper(II)-peptides that could mimic the active site of laccase. The amino acids surrounding the active site of laccases are chemically bonded to four copper ions of three different types (Cu^+^, Cu-O-Cu and Cu^2+^) that catalyze the one-electron oxidation of aromatic and cyclic substrates while reducing oxygen to two molecules of water through a redox reaction [11].

With this in mind, in this work, copper(II)-peptides mimicking the active site of laccase from *Trametes versicolor* were synthesized, characterized and used to catalyze oxidation and polymerization to degrade the aromatic and cyclic PhACs. Copper(II)-peptides are advantageous in terms of cost and stability as the copper in the short chain peptide forms additional stronger ionic and covalent bonds compared to hydrogen bonds in enzymes [12, 13]. Peptides of longer chain are favoured for copper(II) binding compared to di- and tri-peptides as it needs to bind to more than one-metal binding amino acid such as histidine, aspartic acid, glutamic acid, cysteine and methionine to increase the stability of the complex [12]. Copper(II)–peptides have been used as catalysts in asymmetric reactions and have been reported to have good catalytic activity [14]. Up to now, there have not been any copper-peptides reported as catalyst for oxidation reactions. It was hoped that this work benefits environmental and medical industries on the use of copper(II)-peptides in removal of PhACs from drinking water sources, thus addressing one of the biggest environmental threats the world faces today.

## Materials and methods

### Materials

All chemicals and solvents used in these experiments were analytical grade reagents and were used without further purification. The amino acids and HCTU were purchased from GL Biochem (Shanghai) Ltd. Organic solvents were purchased from J.T. Baker, Fisher Scientific and Merck. Water used for the synthesis of copper(II)-peptides (Cu-peptides) and as the aqueous media for the homogeneous catalysis was purified using an Ultrapure Merck Millipore system (resistivity of 18 MΩ.cm at room temperature).

### Physicochemical characterization

High-performance liquid chromatography was carried out using a Waters HPLC (Binary HPLC pump 1525 and UV-Waters 2489) employing solvent gradient elution (deionized water with 0.1% trifluoroacetic acid (TFA) and acetonitrile with 0.1% TFA) with 0.5 mL/min flowing through reversed phase C_18_ column XBridge (4.6 x 250 mm). Liquid chromatography-mass spectroscopic (LC-MS) analyses were conducted using an Agilent 1290 Infinity LC system coupled to Agilent 6520 accurate-mass Q-TOF mass spectrometer with dual ESI source fitted with Agilent Zorbax 300SB-C18 Narrow-Bore RR column (2.1 x 100 mm x 3.5 μm). Experiments were run using the same gradient system and flow rate used for HPLC.

Melting points were measured using a digital melting point apparatus IA 9000 Electrothermal (Cole-Parmer) using glass capillary tubes. The powdered samples were viewed under eye-piece and triplicate readings of melting points for each samples were recorded. The average values are reported herein. Plastic plates used for Thin layer chromatography (TLC) was done using Merck pre-coated plates (silica gel 60 F_254_, 0.2 mm). NMR spectra of peptides and their copper complexes (90% KH_2_PO_4_ buffer and 10% D_2_O) were recorded using an NMR Bruker 700 MHz spectrometer. 1D NMR analysis was conducted at pH 6 and 25°C while 2D NMR were conducted at pH 6 and 4°C (to minimize the rapid exchange of protons in the sample tubes). The spectra were interpreted using TopSpin NMR and Collaborative Computational Project-NMR (CcpNMR) software. The PhACs and their oxidized products were analyzed using Shimadzu gas chromatography equipped with a HP-5-MS (30 m x 0.25 mm x 0.25 μm) column with helium gas flowing at a rate of 0.8 mL/min for 25 minutes. The temperature was programmed from 50°C to 300°C at 10°C/min. All compounds were characterized using Shimadzu mass spectrometry under a full scan mode of 40–500 amu.

### Design and synthesis of peptides

**Fig 1:**
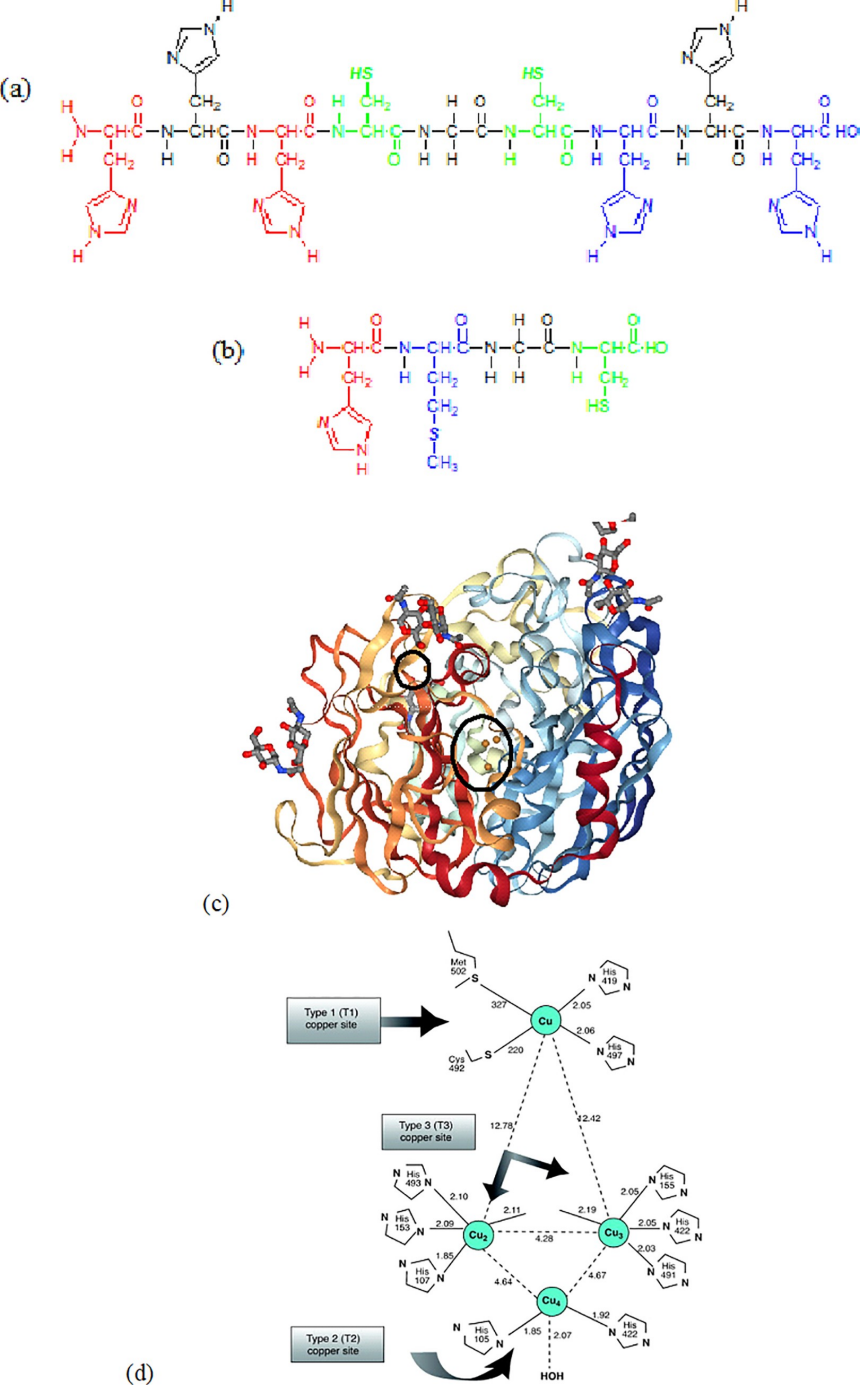
Structure of Np (a) and Tp (b) Laccase from *Trametes versicolor* (pdb: 1KYA) (c) and mononuclear active site highlighting To sequence while trinuclear site highlighting Np sequence with a slight modification of Cys-Gly-Cis in the centre (d). * Red, green and blue: possible binding sites of Cu^2+^ with C = N, C-S and amide (-CONH-).

The nonapeptide (Np) with the amino acid sequence His-His-His-Cys-Gly-Cys-His-His-His was derived from laccase *Trametes versicolor* active site (pdb: 1KYA) which was surrounded by trinuclear and mononuclear copper centres [15]. The nonapeptide was shortened and modified to a tetrapeptide (Tp) having the amino acid sequence His-Met-Gly-Cys as shown in Fig 1. The pdb file of laccase was attained from RCSB PDB (https://www.rcsb.org/) and the active site was studied using MetalPDB (http://metalweb.cerm.unifi.it/search/pdb/1KYA/). The peptide sequences were modified using PEP-FOLD (https://bioserv.rpbs.univ-paris-diderot.fr/services/PEP-FOLD/) and Lomets (https://zhanglab.ccmb.med.umich.edu/LOMETS/) and the resulting pdb was viewed using PyMOL. The general synthesis of Tp which followed procedures reported in our previous work [14] is given in the (S1 Scheme in S1 File).

### Synthesis of copper(II)-peptides

The copper(II)-peptides (Table 1) were synthesized using copper(II) acetate and copper(II) sulphate following procedures outlined in our previously reported work [14]. Aqueous copper(II) solution (1.6 mmol, 5 mL) was added dropwise to the pre-prepared peptide aqueous solution (0.8 mmol, 5 mL). The solution was stirred at 125 rpm and maintained at 40°C and pH 6 for 2 hours until a colour change and/or precipitation took place. The crude precipitates formed were crystallized.

**Table 1:** List of catalysts used for xenobiotic degradation of PhACs.

Peptides/Peptides-Cu(II)	Cu-peptide molar ratio	Abbreviation
Np	-	Np
Np-CuSO_4_	1:4	NpCuS 14
Np-CuSO_4_	4:1	NpCuS 41
Np-Cu(CH_3_COO)_2_	1:2	NpCuC 12
Np-Cu(CH_3_COO)_2_	1:4	NpCuC 14
Np-Cu(CH_3_COO)_2_	4:1	NpCuC 41
Tp	-	Tp
Tp-CuSO_4_	1:2	TpCuS 12

### Xenobiotic degradation of PhACs by oxidation

Catalysts (peptides and Cu-peptides) were added in 30% hydrogen peroxide in 1: 0.1: 0.1 molar ratio of substrate: catalyst: H_2_O_2_. The substrate was added to the catalyst solution and was stirred at 125 rpm at the optimized conditions (30°C, 12 hours) in the absence of light [16].

Catalyst was separated from the degraded products by acidifying the final solution with hydrochloric acid (0.1 mL, 1 M) followed by the addition of magnesium sulphate (2 g) and sodium chloride (0.5 g). The mixture was manually shaken until all the solid dissolved. The solution was vortexed for two minutes after addition of 10 mL ethyl acetate. The organic layer (products) was separated from the aqueous layer (catalyst layer) by decanting. The organic layer was dried using anhydrous sodium sulphate. The mixture was filtered and washed with ethyl acetate (5 mL) into a pre-weighed, dry glass vial. The solvent was evaporated using a stream of dry nitrogen for 5 minutes [17].

For chromatographic analysis, the compounds were dissolved in 50% v/v deionized water and methanol with 0.1% formic acid and sonicated for five minutes. The product was filtered through PTFE syringe filters (0.25 mm x 0.45 μm) and transferred to clean, dry gas chromatography vials [18].

## Results and discussion

### Physicochemical characterization

The physical characteristics of the solid apo-peptides and their copper complexes are given in Table 2. The sharp melting points observed indicate that these compounds were pure. The yields of copper(II)-peptides synthesized in this work are comparable to those previously reported for tetrapeptides used as catalysts for aldol reactions [14].

**Table 2:** Physical characteristics and yields of solid peptides and their Cu complexes.

Peptides/Cu-peptides	Physical appearance	% Yield	Melting point/°C	Solvent solubility	pH
Np	pale yellow powder	1580 mg (33.6%)	170.2 ± 0.2°C	Water (5 mL + 50 μL TFA)	2.61
NpCuC 12	dark green crystals	40.2 mg (29.5%)	230 ± 0.0°C	Hot ethanol (5 mL)	3.99
Tp	white powder	1480 mg (40.0%)	152.5 ± 0.1°C	Water (5 mL + 50 μL TFA)	3.17
TpCuC 12	turquoise crystals	21.5 mg (25.4%)	258.0 ± 0.0°C (decompose)	Hot ethanol (5 mL)	5.12

*m: melted, **d**: decomposed.

*Np: Nonapeptide HHHCGCHHH, **Tp:** Tetrapeptide HMGC.

**Fig 2:**
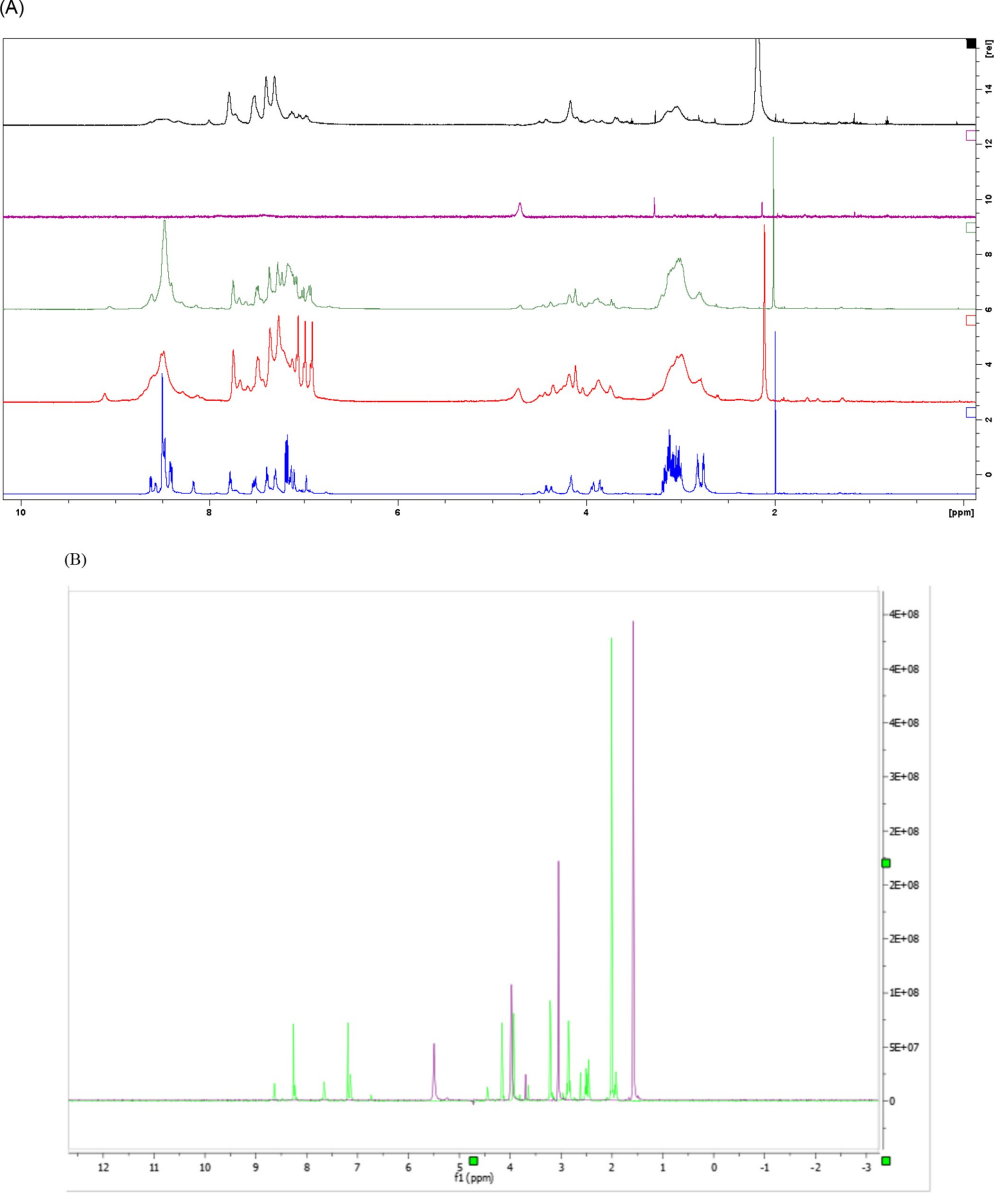
1D ^1^H NMR spectra of peptides and their Cu-peptides at pH 6 and 25°C. (a) Apo-Np, NpCuC 41 & 14 and NpCuS 41 & 14. *Blue: Apo-Np, **Red**: NpCuS 14, **Green**: NpCuS 41, **Purple**: NpCuC 41, **Black**: NpCuC 14. Spectra are on a scale of 16. *NMR of NpCuC 12 was not recorded as it precipitated with the deuterated solvents.(b) Apo-Tp (green) and TpCuS 12 (purple).

The remaining copper-peptides (Np-CuC 14, 41, Np-CuS and Tp-CuS) did not precipitate and were aqueous upon synthesis; hence physical characterizations could not be conducted. The apo-peptides were analysed using HPLC-UV and LC-MS while the copper(II)-peptides were characterized by ^1^D NMR. These results are presented in Table 4 and the NMR spectrum is shown under Fig 2. ^2^D NMR spectra are reproduced in (S1 Table and S1 Fig in S1 File).

### HPLC & LCMS analysis of peptides

The purity of each peptide prepared using solid phase peptide synthesis (SPPS) was determined using HPLC -UV fitted with a C_18_ reverse phase column employing the optimised gradient system. Trifluoroacetic acid (TFA) was added to the mobile phase as the ion-pairing reagent to mitigate strong interactions between the silica packing and the peptide which could obstruct the flow of the peptide through the column [19]. The purity calculated for each peptide as its percentage of peak area is given in Table 3. The purities of the peptides were similar when analysed by both HPLC and LCMS. The charge on each peptide was calculated to be -1 employing the *m*/*z* charge ratio, making the peptides suitable ligands for binding with copper ions. The calculated molecular masses agreed with those determined using mass spectrometry.

**Table 3:** HPLC-UV and LC-MS analytical results.

Peptides	R_T_/min	Purity/%	Molecular mass/g mol^-1^	m/z	Charge
Np	9.378	93.1	1325.42 (1325.45)	1326.50	-1
Tp	4.913	9.2	445.16	444.15	-1
	5.660	78.3	445.16(445.57)	444.15	

**m/z** mass to charge ratio, **R**_**T**_ is the retention time, (Theoretical mass).

### NMR analyses of peptides and Cu-peptides

Nuclear magnetic resonance (NMR) spectroscopy was used to determine the peptide sequences based on the proton-proton and carbon-proton interactions in the amino acids. It can also be used to predict the structures of oligopeptides and long chain peptides (sequence greater than 15 amino acids) when the degree of flexibility and the exchange of proton-proton interchange over space (NOESY) is reduced due to the stable rigidity of the structure [20]. The peptides and Cu-peptides were characterized by both 1D and 2D NMR involving proton NMR, TOCSY ^1^H-^1^H and HSQC ^1^H-^13^C.

The peptides sequences were confirmed by NMR using TOCSY and HSQC. The data is given in Table 4 and S1 Table in S1 File respectively. TOCSY is used to identify protons through bond spin-spin couplings within a spin system. It detects protons within 3–5 bond distances [21]. Heteronuclear single quantum coherence (HSQC) is an NMR experiment used to determine proton-proton single bond correlations [22]. The chemical shifts of protons (F2 axis) plotted against the ^13^C chemical shifts (F1 axis) of directly attached carbons determined through ^1^J_CH_ coupling are shown in S5C Fig in S1 File.

**Table 4:** ^1^H NMR chemical shifts of peptides.

Amino acid	Theoretical**	Np (HHHCGCHHH)*	Tp (HMGC)*
	δ/ppm	δ/ppm (T, Int)	δ/ppm (T, Int)
His 1	8.26, 7.82, 7.17, 4.62, 3.16, 3.10	7.39(t,3H), 7.13 (m,6H), 4.38 (d,1H), 3.19–3.00 (m,12H)	7.66 (t,1H), 7.17 (d,3H), 4.16 (t,2H), 3.21 (d,2H)
His 2		8.40 (s,1H), 7.30 (t,2H), 7.10 (m,6H), 4.37 (d,1H), 3.19–3.00 (m,12H)	-
His 3		8.48 (d,1H), 7.78 (t,2H), 7.19 (m,6H), 4.51(m,1H), 3.19–3.00 (m,12H)	
His 7		8.50 (d,1H), 7.71 (m,1H), 7.18 (m,6H), 4.50 (m,1H), 3.19–3.00 (m,12H)	
His 8		8.41(s,1H), 7.51 (m,1H), 7.16 (m,6H), 4.42 (m,1H), 3.19–3.00 (m,12H)	
His 9		8.41(s,2H), 7.52 (m,1H), 7.17 (m,6H), 4.43 (m,1H), 3.19–3.00 (m,12H)	
Met	8.26, 4.41, 2.36, 2.33,	-	8.26 (m,2H), 4.41 (t,2H), 2.49 (d,4H), 1.92 (s,2H)
Gly	8.33, 3.96, 3.89	8.17 (d,1H), 3.92 (dd,1H), 3.85 (dd,1H)	8.26 (m,2H), 3.93 (d,2H)
Cys 4	8.38, 4.68, 3.16, 3,10, 2.01	8.57 (d,1H), 4.09 (s,3H), 2.81 (dd,5H), 2.74 (dd,4H), 1.95 (s,1H)	8.63 (t,1H), 4.44 (m,1H), 2.84 (dd,5H), 2.00 (d,1H)
Cys 6		8.63 (d,1H), 4.16 (s,3H), 2.82 (dd,5H), 2.75 (dd,4H), 2.08 (s,1H)	-

*: attained ** http://www.bmrb.wisc.edu/ref_info/statful.htm (Theoretical NMR values) [23].

His1-3 and His 7–9: Specific assignments to differentiate between the histidine could not be carried out in Nonapeptide. The chemical shifts corresponding to each histidine are based on the spin system of different chemical environment between neighbouring amino acids.

The differences between different molar ratios of copper(II) acetate and sulphate bound to the nonapeptide containing 21N with C = N potential binding sites are shown Fig 2A. When copper(II) acetate [5 mM] was added to the peptides, the signals detected for the aromatic protons from the imidazole group in histidine at ~δ = 7–8 ppm disappeared for Np-Cu(CH_3_COO)_2_ (1:4 molar ratio) due to the large presence of paramagnetic copper(II) ions. The number of protons increases at ~δ = 7–8 ppm as the amount of peptide increases as seen in the 4:1 molar ratio (Np:Cu^2+^) spectrum. Additional signals were also observed in the spectrum due to the paramagnetism of Cu^2+^. Aliphatic protons in the glycine and cysteine groups (H_α_-2H δ~4 ppm, H_β_-1H δ~3–4 ppm, H_γ_-1H δ~2 ppm) were observed evidenced by the integration for each peak in the spectrum. During the preparation of Np-Cu for NMR analysis, Np-CuC 12 precipitated upon the addition of deuterated water in the NMR tube, hence NMR analysis of Np-CuC 12 was not conducted. This could be due to the formation of Np-CuC 12 crystals at 4°C at which the samples were stored for the analysis.

**Fig 3:**
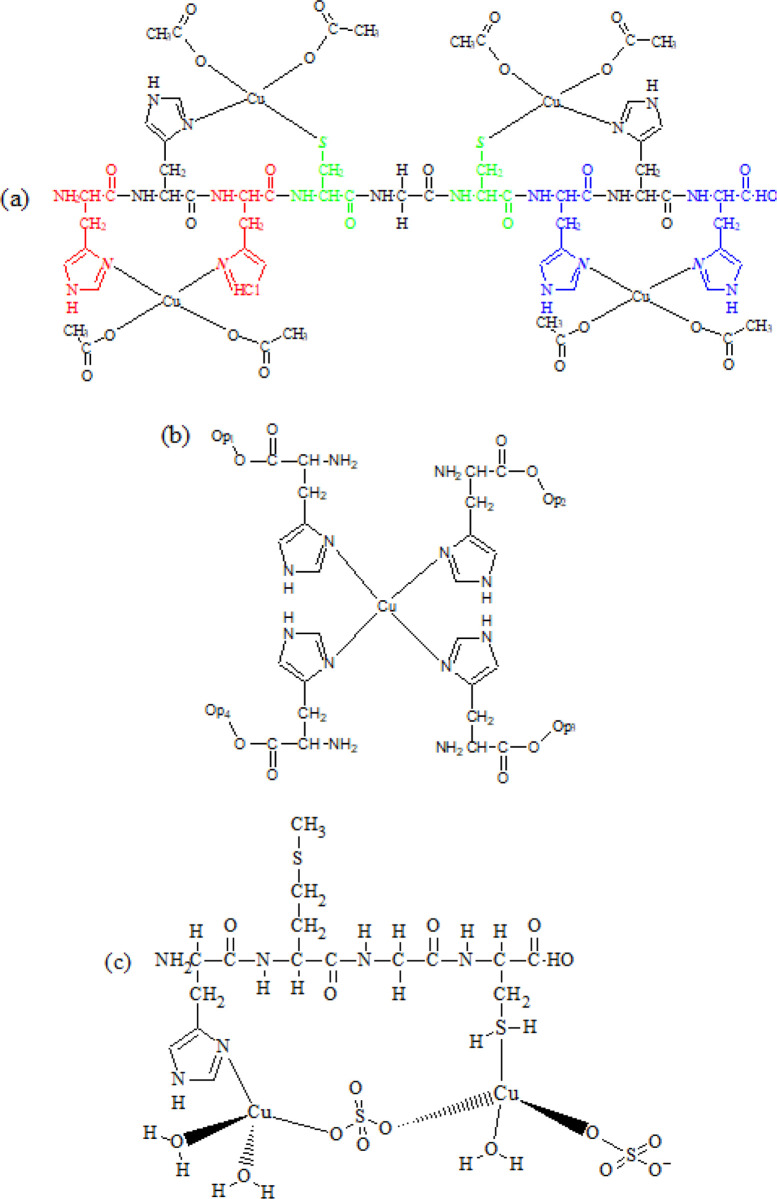
NMR spectra suggest square planar geometry for (a) Np-Cu(CH_3_COO)_2_ in 1:4 ratio, (b) Np-Cu(CH_3_COO)_2_ 4:1 ratio; preferred due to steric hindrance of side chains and (c) tetrahedral geometry for Tp-CuSO_4_ 1:2 ratio with bridging sulphate ligand. *Op_1-4_ the remaining 8 amino acids of the nonapeptide (Octapeptide).

That no signals were observed for the amide protons (δ = 8-9ppm) indicating that the 4 moles of Cu^2+^ from Cu(CH_3_COO)_2_ were bound to the nitrogen (C = N) in the imidazole ring of histidine, sulphur (C = S) in the cysteine and to the amide nitrogen can be taken as support for square planar geometry (Fig 3). A similar conclusion stating that Cu^2+^ ions formed square planar geometry when bound to 3 different peptides with sequences AH, AHH and AAH separately was reported by Gonzalez *et al*. [24]. Copper complexes are known to form tetrahedral geometry over octahedral geometry for Tp-CuS complexes where copper(II) is bound to the same ligands. Also, due to steric hindrance, Np-CuC prefers a square planar geometry following the theory of Jahn-Teller distortion as shown in Fig 3 [24]. The confirmation of structure through X-ray diffractometry (XRD) could not be done. Numerous attempts to grow crystals using various techniques gave crystals that were not suitable for single crystal XRD analysis. This was because of their small size (as compared to copper-proteins) and their high degree flexibility that forming crystals is difficult hence precluding structure determination via XRD [25].

The effects of different counter ions were also investigated from NMR spectra Fig 2A. Sulphate ion (SO_4_)^2-^, though a good leaving group bound to Cu^2+^ stronger than acetate (CH_3_COO)^-^, leaving Cu^2+^ with fewer empty orbitals to accept electrons from nitrogen (C = N) in the imidazole ring of histidine. Fewer H_δ_ and H_ε_ belonging to histidine were observed in Fig 2A for Np-CuC compared to the Np-CuS (4:1 and 1:4 molar ratios). When one mol of Tp bound to two moles of CuSO_4_ to give TpCuS 12, no proton signals were seen at δ = 7–8 ppm, indicating that the Cu^2+^ ions were bound to histidine. A possible hypothesis to explain the phenomena is that the second Cu^2+^ bound to cysteine at position 4 because the proton S-H doublet peak of cysteine shifted upfield and the H_α_ of cysteine was downshifted indicating the presence of electronegative sulphate ions. TpCuS 12 was predicted to be tetrahedral in solution due to the presence of different ligands bound to copper(II) namely 2 H_2_O, 1 N/S of Tp and the other a bridging sulphate ion through S-O^-^). A similar finding was reported by Hashim [21] who predicted the geometries of different Cu^2+^ bound to several histidine and cysteine while different anions (sulphate and nitrate) were still attached to copper(II) [21].

### Xenobiotics degradation of PhACs

Oxidative fungal enzymes such as laccase, also known as green catalyst, have been used to catalyze the degradation of recalcitrant pharmaceuticals in the environment. Even though, laccases are not as effective as peroxidases, these lignolytic enzymes are preferred since the production of radicals that depletes the ozone layer can be avoided [26]. However, extracting these enzymes is time-consuming, requires plenty of raw materials and utilizes specialized techniques due to their sensitivity to changes in temperature and pH. They are stable from ~20 to 40°C and at pH = 7. Over the past few years, researchers have studied alternative methods such as using biocatalysts that mimic the activity of these enzymes.

In this work, copper(II)-peptides were designed to mimic the active sites in laccase from *Trametes versicolor*. It was expected that they would show high oxidation activity and be able to degrade selected PhACs [3]. Their catalytic activity was evaluated and compared with that of apo-peptides (Table 5) as well as with laccase from *Trametes versicolor* (positive control), H_2_O_2_ and a mixture of laccase and H_2_O_2_, where H_2_O_2_ acts as both the oxygen support and to mediate the activity of laccase (Table 6). In this experiment, only solid catalysts were used. Aqueous-based NpCuC and NpCuS (14 and 41) were not used as water can affect the degradation of organic PhACs.

**Table 5:** Xenobiotics degradation of PhACs using apo-peptides and their Cu-peptides.

Catalysts Substrate	Tp		Tp-CuS (1:2)		Np-CuC (1:2)	
	%C	Products	%C	Products	%C	Products
*o*-cresol	0	No activity	**83.9**	2-hydroxy-2-methylbutanoic acid (17.8%), 2,2-dimethoxybutane (59.6%) Methyl-2-hydroxyisocaproate (6.48%)	0	No activity
*m*-cresol	0	No activity	**0.26**	2,2-dimethoxybutane (0.10%), 3,4-dimethylphenol (0.16%)	0	No activity
*p*-cresol	0	No activity	0.10	2,2-dimethoxybutane	**3.06**	m-phenoxybenzoic acid (0.56%) 1,4-dimethyl-7-(1-methylethyl)-azulen-2-ol (1.65%) ketones/diones (0.85%)
2,4-dichlorophenol	0	No activity	**5.85**	2,2-dimethoxybutane	0	No activity
Salbutamol	**96.8**	Benzoic acid	52.9	2,2-dimethoxybutane	**92.4**	3-(4-methoxyphenoxy)-1,2-propanediol
Erythromycin	**57.4**	1,3,6-Trioxocane (33.2%), 1,4-Dioxane (24.0%)	49.7	Tetramethyl-D-(+)-xylose (8.07%), Ethyl decanoate (3.22%), 1,3:2,5-dimethylene-4-methyl-d-rhamnitol (38.4%)	34.7	N,N`-dimethyl-urea (19.6%) 1,4-Dioxane (15.1%)
Ibuprofen	29.9	N-propxycarbonyl-butyl-I-valine ester	**35.1**	α-methyl-4-(2-methylpropyl)-benzeneacetaldehyde	3.57	Ibuprofen methyl ester (2.05%) α-methyl-4-(2-methylpropyl)-benzeneacetaldehyde (1.52%)

*Apo-Np was an ineffective catalyst as no activity or low activity (<1%) was detected by GC-MS.

*Since NpCuC 14&41 and NpCuS 14&41 were in aqueous solution, they were not used for this organic degradation as water affects the reaction. Np, Tp, NpCuC 12 and TpCuS 12 were solid catalysts.

*No oxidation activity was observed for medications diphenylhydrazine (Uphadyl Forte), insulin (Insuman Rapid& Basal), diclofenac, Celebrex, orphenadol, heparinol and cetrizine.

%C: % conversion/degradation.

**Table 6:** Xenobiotics degradation of PhACs using laccase, H_2_O_2_, laccase & H_2_O_2_.

No	Substrate	Oxidized products (% conversion, m/z in amu)		
		Laccase	Laccase + H_2_O_2_	H_2_O_2_
1	o-cresol	Benzoic acid (2.4%, 123.12)	2-chloroethyl-3-methylphenyl carbonic acid ester (3.6%, 214.091)	2-methyl-p-benzoquinone (1.8%, 122.037)
2	Salbutamol	m-cresol (7.5%, 108.06) p-cresol (92.5%, 108.06)	Benzoic acid (85.1%, 205.074)	o-cresol (4.5%) m-cresol (95.5%)
3	Uphadyl Forte	Benzoic acid (9.7%, 122.04)	Methylparaben (54.4%, 152.047)	Benzoic acid (7.1%) 3-methyl-1,2-benzenediol (8.4%, 124.052) Methylparaben (8.8%)
4	Erythromycin	3-(1-Methoxy-1-methylethoxy)-2-methylpropionic acid (44.6%, 172.146) 14,15-diethyl-bicyclo[10.4.0]hexadecane (30.7%, 249.079)	methyl 2,3,4-tri-O-methyl-β-D-xylopyranoside (14.0%, 204.063) 3-(1-Methoxy-1-methylethoxy)-2-methylpropionic acid (20.0%)	3-(1-Methoxy-1-methylethoxy)-2-methylpropionic acid (4.5%)
5	Insuman Rapid	Oleanitrile (65.1%, 476.129)	4-chloro-m-cresol (39.8%, 142.019)	4-chloro-m-cresol (83.5%)
6	Insuman Basal	1,1,3,3,5,5,7,7,9,9,11,11-dodecamethyl-hexosiloxane (100%, 250.121)	4-chloro-m-cresol (48.6%)	4-chloro-m-cresol (25.3%)
7	Cataflam/ Diclofenac	2-ethylbutyl octyl-2,6-pyridinedicarboxylic acid ester (9.0%, 276.101)	1,3-dihydro-2H-Indol-2-one (97.5%, 277.006)	1,3-dihydro-2H-Indol-2-one (82.0%)
8	Celebrex	13-(Z)-Docosenamide (1.3%, 337.334)	5-Methylbenzimidazo[2,1-a]phthalazine (56.0%, 302.153)	5-Methylbenzimidazo[2,1-a]phthalazine (66.1%)
9	Ibuprofen	6-amino-2-(4-methylphenyl)- naphthalimide (100%, 302.106)	1-(p-fluorophenyl)-anthraquinone (100%, 302.065)	1-(p-fluorophenyl)anthraquinone (100%)
10	Orphenadol	2-(2,4-dinitrophenylhydrazono) propionic acid (16.3%, 268.044)	Methyl hexadecanoic acid ester (0.4%, 270.256)	Methyl hexadecanoic acid ester (0.4%)
11	Heparinol	Benzyl Alcohol (92.2%, 108.058) 6-Octadecenoic acid (1.4%, 252.282)	Benzyl Alcohol (92.2%) Dimethyl acetal benzaldehyde (2.4%, 152.084)	Benzyl Alcohol (92.8%) Dimethyl acetal benzaldehyde (3.1%)
12	Cetrizine	Methylparaben (45.6%, 152.047) Chlorcyclizine (1.7%, 300.139)	Methylparaben (31.7%) chlorcyclizine (1.2%)	Methylparaben (23.6%) chlorcyclizine (1.1%)

* m/z values are not repeated for the same products.

The highest activities observed for oxidation of these PhACs using the apo-peptide and Cu(II)-peptide catalysts were attained conditioning the catalysts at the optimal temperature, pH, media (for the heterogeneous catalysis), O_2_ source from 30% H_2_O_2_, time and stirring conditions in the absence of light. The heterogeneous catalysts oxidized pharmaceuticals containing phenol groups such as cresols, 2,4-dichlorophenol and Salbutamol to 2,2-dimethoxybutane. The structures of the final oxidation products obtained from the reaction of PhACs catalyzed by the positive controls, apo-peptides and Cu-peptides are given in Table 2 in (S2 Table in S1 File). Cresols (*o-*, *m-*, *p-*) were not oxidized when apo-peptides were used to catalyze the reaction but when copper(II)-peptides were used, *o*-cresol was oxidized to 2-hydroxy-2-methylbutanoic acid, methyl-2-hydroxyisocaproate and degraded to 2,2-dimethoxybutane (major product). The aliphatic products were readily degraded to CO_2_ and H_2_O at high temperatures. These observations indicate that copper(II) plays an important role in the oxidation of cresols, was however the ttetrapeptide oxidized 97% of Salbutamol to benzoic acid while only 53% was degraded to 2,2-dimethoxybutane by Tp-CuS. Salbutamol was not oxidized by Np while Np-Cu converted 92% of Salbutamol to 3-(4-methoxyphenoxy)-1,2-propanediol. The complex chemical structures usually incorporating ribose sugars, thus Erythromycin, an antibiotic having a comparatively complex structure was degraded into multiple chemical compounds. In contrast with cresols, Erythromycin exhibited higher oxidation activity when catalyzed by Tp rather than by its Cu complex.

Laccase combined with H_2_O_2_ produced higher oxidation activity than the individual catalysts (Table 6) for the PhACs studied in this work, laccase alone oxidized 65% of insulin (Insuman Rapid) to 2,4,6-trichlorophenyl tridecyl fumaric acid ester. Tran *et al*. [16] reported the full oxidation of diclofenac using crude laccase obtained from *Trametes versicolor* in the presence of MnSO_4_ and H_2_O_2_ following the same method outlined in the methodology [16]. Similar observations were recorded in this research where 98% of diclofenac was oxidized to 1,3-dihydro-2H-indol-2-one using laccase with H_2_O_2_. In 2012, Rodarte-Morales *et al*. reported that full oxidation of diclofenac and ibuprofen using commercial laccase with mediators (ABTS & HBT) required 12 hours while in the absence of mediators, three weeks were required to fully oxidize the same substrates [17]. Meanwhile, a partial degradation of ibuprofen was reported by Wen et al. [27] for the reaction catalyzed by lignin peroxidase and laccase. In this work, ibuprofen was completely polymerized to 6-amino-2-(4-methylphenyl)- naphthalimide when using laccase and to 1-(p-fluorophenyl)-anthraquinone when laccase + H_2_O_2_ or H_2_O_2_ alone were used (Table 6) [27].

**Scheme 1:**
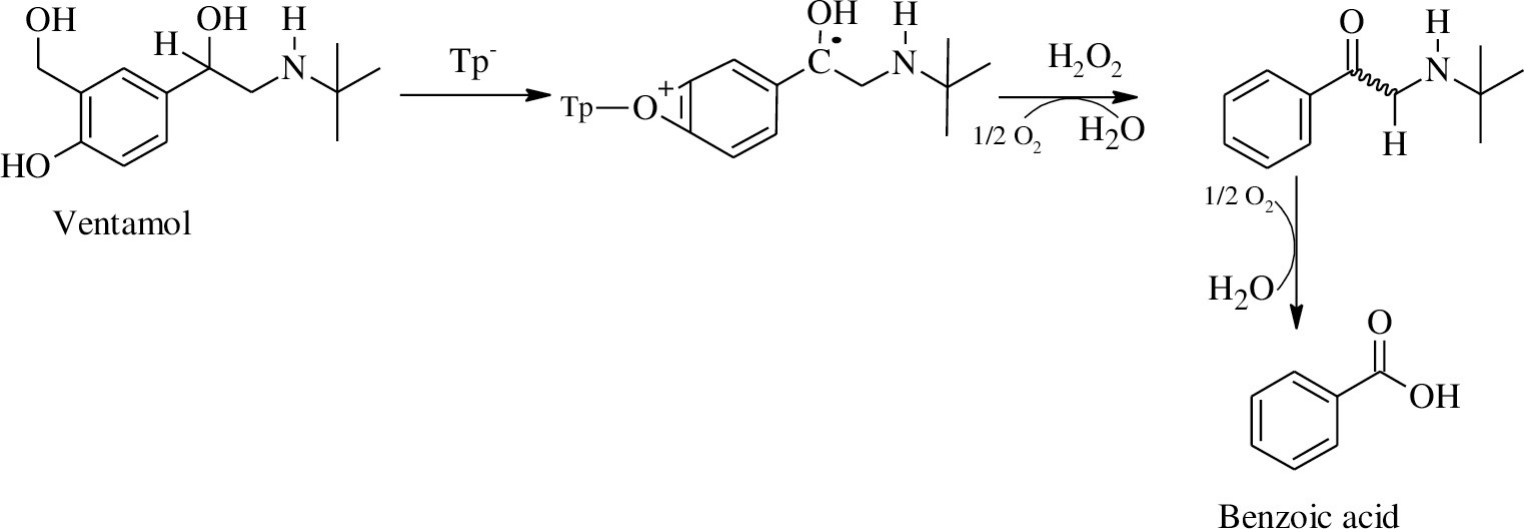
Proposed mechanism for the oxidation of Salbutamol catalyzed by Tp.

**Scheme 2:**
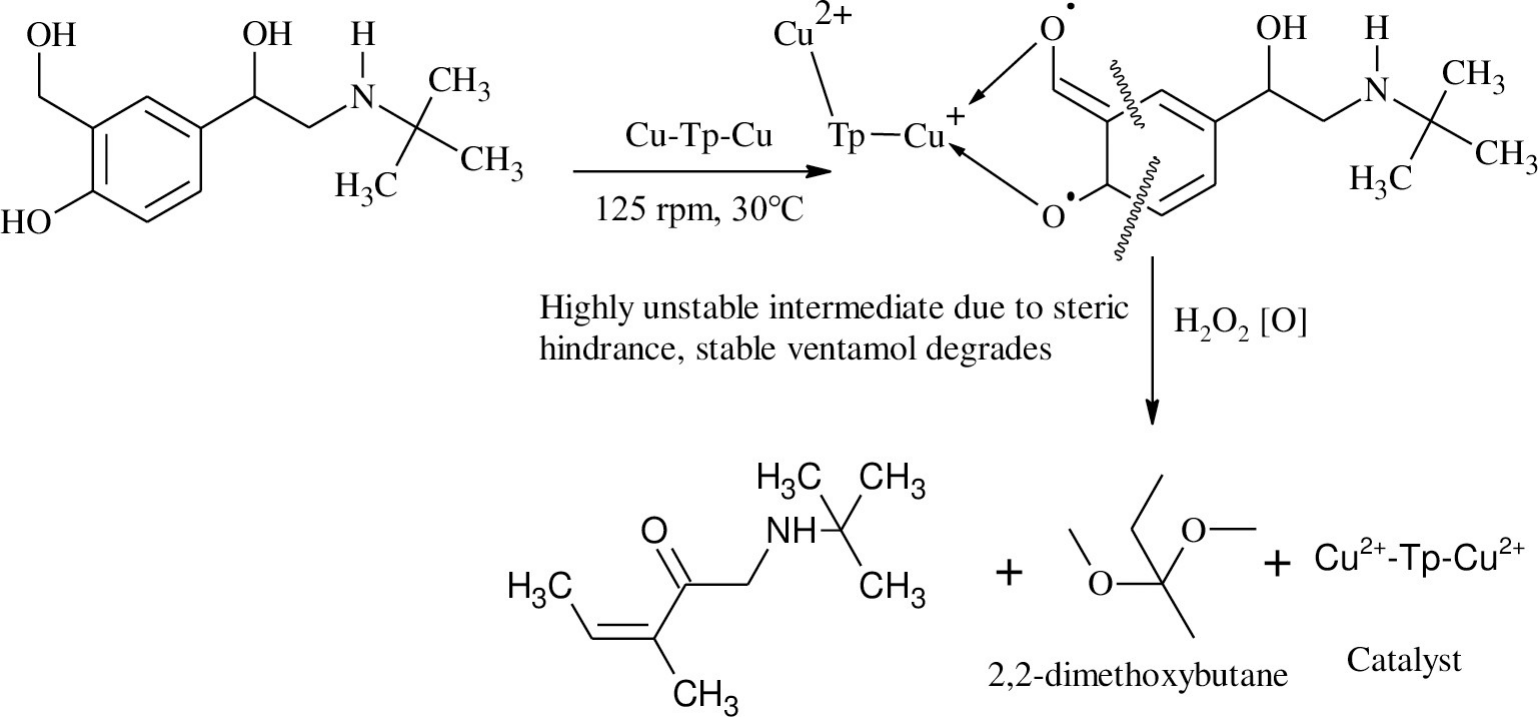
Proposed mechanism for the oxidation of Salbutamol catalyzed by Cu^2+^-Tp-Cu^2+^.

Laccase-catalyzed oxidation of erythromycin and ibuprofen showed higher activity than the peptides and Cu-peptides. However, for o-cresol and ventamol, the peptides and Cu-peptides catalyzed reaction yielded a higher percentage of oxidized products as compared to laccase. Interestingly, different catalysts involved in the degradation of PhACs yielded different products suggesting that the catalysts bound to the substrates differently leading to different intermediates and hence different products. The proposed catalytic mechanisms of the peptides and copper(II)-peptides in this work are shown in Schemes 1 and 2 respectively. Ventamol (Salbutamol) was chosen as a representative of all PhACs as the highest oxidation activity was reported for the Tp and Cu-peptide catalyzed reaction. The mechanism was proposed after comparing the results of similar oxidation studies of cresols using peptides [28] and copper(II) salts [29]. To date, there are no reports on the oxidation of PhACs using copper-peptides; this is also the first of its kind for xenobiotic degradation studies of the mentioned PhACs reported herein.

## Conclusions

Laccase, an oxidative copper(II)-enzyme, was used as the basis for the degradation of phenol-targeting pharmaceutically active compounds in this research. Copper(II)-peptides designed to mimic the structure were synthesized and characterized. The apo-peptides and their Cu-peptides enhance the oxidation activity of laccase and provided an environmentally-friendly alternative using fewer chemicals and producing less harmful by-products. Herein, apo-Tp was reported to have the highest activity in degrading salbutamol and erythromycin while Tp-CuS 12 was observed to be the best catalyst in degrading the cresols, 2,4-dichlorophenol and ibuprofen. Although laccase reported some activity in the degradation of insulin, diclofenac, celebrex, orphenadol, heparinol and cetirizine, the degraded products formed from these PhACs are cresols which were degraded using Tp-CuS 12. Being the smallest organometallic catalyst among the others reported herein, TpCuS 12 is the best catalyst and shows promising potential for use in environmental applications.

## Supporting information

S1 File (DOCX)
