# Supplementary material for: Imidazole-rich copper peptides as catalysts in xenobiotic degradation
Source: PLoS One. 2020 Nov 4;15(11):e0238147. doi: 10.1371/journal.pone.0238147 (PMC7641441; doi:10.1371/journal.pone.0238147)
Supplement: S1 File — (DOCX) [file pone.0238147.s001.docx]

| List | Caption |
| --- | --- |
| S1 Table | 2D NMR TOCSY & HSQC of peptides |
| S2 Table | Molecular structure of PhACs and their degraded products |
| S3 Table | Raw data obtained from GCMS spectra for the mentioned PhACs and catalysts |
| S1 Scheme | Solid phase peptide synthesis of Tp |
| S1 Fig | 2D NMR TOCSY and HSQC of peptides overlapped with their Cu-peptides. |

**S1 Table. ^2^D NMR TOCSY & HSQC of Np**

| No. | Theoretical [17] | | Obtained | | Assignment | Amino acid |
| --- | --- | --- | --- | --- | --- | --- |
|  | f1/ppm | f2/ppm | f1/ppm | f2/ppm |  |  |
| 1 | 30.32 | 4.62 | 25.22 | 4.37 | Cβ, Hα | His |
| 2 |  |  | 25.39 | 4.42 | Cβ, Hα |  |
| 3 |  |  | 25.40 | 4.42 | Cβ, Hα |  |
| 4 |  | 3.16/3.10 | 25.47 | 2.82 | Cβ, Hβ1/Hβ2 |  |
| 5 |  |  | 25.50 | 2.75 |  |  |
| 6 |  | 4.62 | 25.63 | 4.18 | Cβ, Hα |  |
| 7 |  | 4.62 | 26.02 | 4.60 | Cβ, Hα |  |
| 8 | 33.25 | 4.68 | 26.31 | 4.50 | Cβ, Hα | Cys |
| 9 |  | 3.16 | 26.22 | 3.13 | Cβ, Hβ1 |  |
| 10 |  | 3.08 | 26.36 | 3.08 | Cβ, Hβ2 |  |
| 11 |  | 2.06 | 20.32 | 1.99 | Cβ, Hγ |  |
| 12 | 45.38 | 8.33 | 42.29 | 8.51 | Cα, H_N_ | Gly |
| 13 |  | 3.96 | 42.31 | 3.93 | Cα, Hα1/Hα2 |  |
| 14 |  | 3.89 | 42.32 | 3.85 |  |  |
| 15 | 56.52 | 4.62 | 46.69 | 4.09 | Cα, Hα | His |
| 16 |  |  | 46.78 | 4.16 |  |  |
| 17 |  |  | 52.47 | 4.56 |  |  |
| 18 | 56.52 | 8.26 | 52.50 | 8.43 | Cα, H_N_ | His |
| 19 |  |  | 52.63 | 8.40 | Cα, H_N_ | His |
| 20 | 56.52 | 3.10 | 52.79 | 3.06 | Cα, Hβ1/Hβ2 | His |
| 21 |  | 3.16 | 53.06 | 3.18 |  |  |
| 22 | 56.52 | 8.26 | 53.09 | 8.48 | Cα, H_N_ | His |
| 23 |  | 8.26 | 55.41 | 8.17 | Cα, H_N_ | His |
| 24 |  | 7.17 | 53.53 | 7.14 | Cα, Hδ | His |
| 25 |  | 4.62 | 55.41 | 4.37 | Cα, Hα | His |
| 26 |  | 3.10 | 55.42 | 2.75 | Cα, Hβ1/Hβ2 | His |
| 27 |  | 3.16 | 55.56 | 2.81 |  |  |
| 28 |  | 4.62 | 55.59 | 4.42 | Cα, Hα | His |
| 29 |  | 8.26 | 55.63 | 8.42 | Cα, H_N_ | His |
| 30 |  | 4.62 | 66.01 | 4.16 | Cα, Hα | His |
| 31 |  | 4.62 | 66.28 | 4.42 | Cα, Hα | His |
| 32 |  | 4.62 | 66.28 | 4.50 | Cα, Hα | His |
| 33 | 58.04 | 8.38 | 52.26 | 8.62 | Cα, H_N_ | Cys |
| 34 |  | 4.68 | 52.37 | 4.56 | Cα, Hα |  |
| 35 |  | 3.16 | 52.32 | 3.00 | Cα, Hβ1 |  |
| 36 |  | 3.08 | 52.39 | 3.12 | Cα, Hβ2 |  |
| 37 |  | 8.38 | 52.48 | 8.57 | Cα, H_N_ |  |
| 38 | 119.90 | 7.17 | 116.97 | 6.97 | Cδ, Hδ | His |
| 39 |  |  | 117.09 | 7.18 | Cδ, Hδ | His |
| 40 |  |  | 117.14 | 7.10 | Cδ, Hδ | His |
| 41 |  |  | 118.04 | 7.39 | Cδ, Hδ | His |
| 42 |  |  | 120.06 | 7.27 | Cδ, Hδ | His |
| 43 |  |  | 120.08 | 7.30 | Cδ, Hδ | His |
| 44 | 131.31 | 7.82 | 124.76 | 7.51 | Cγ, Hε | His |
| 45 |  |  | 127.41 | 7.51 | Cγ, Hε | His |
| 46 |  |  | 127.47 | 7.45 | Cγ, Hε | His |
| 47 |  |  | 127.90 | 7.78 | Cγ, Hε | His |
| 48 | 137.21 | 8.26 | 133.59 | 8.48 | Cε, H_N_ | His |

**S2 Table. Molecular structure of PhACs and their oxidized products**

| PhACs substrate | Oxidized Products | | | | | | | |
| --- | --- | --- | --- | --- | --- | --- | --- | --- |
| 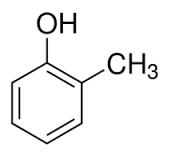  o-cresol | 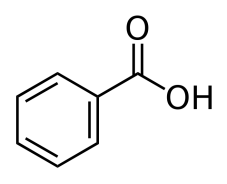  a) Benzoic acid^1^ | b)  2-chloroethyl-3-methylphenyl carbonic acid ester^2^ | | | c)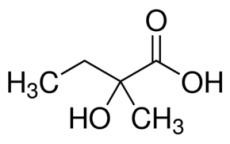  2-Hydroxy-2-methylbutanoic acid^5^ | | | d)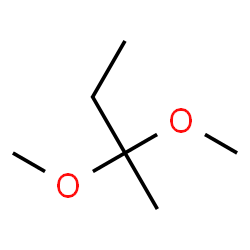  2,2-dimethoxybutane^5^ |
| 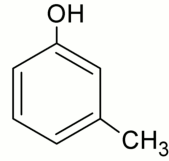  m-cresol | a)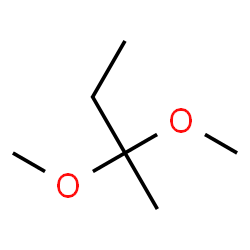  2,2-dimethoxybutane^5^ | | | b)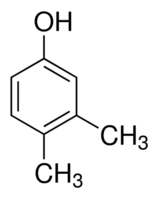  3,4-dimethylphenol^5^ | | | | |
| 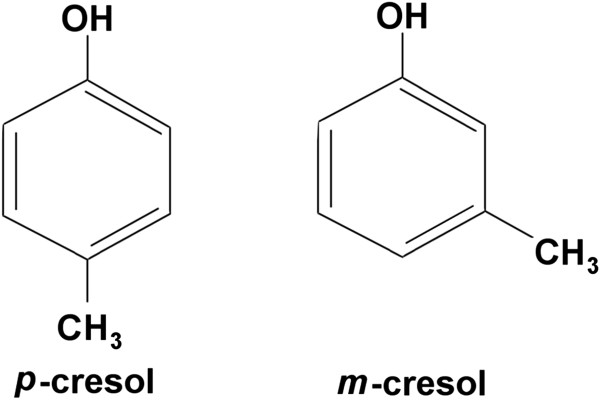  p-cresol | a)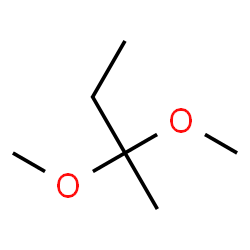  2,2-dimethoxybutane^5^ | | | b)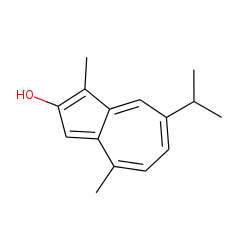  1,4-dimethyl-7-(1-methylethyl)-azulen-2-ol^6^ | | | | |
| 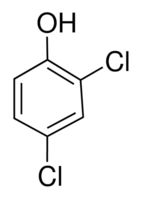  2,4-dichlorophenol | a)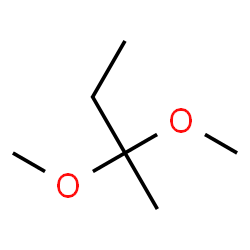  2,2-dimethoxybutane^5^ | | | | | | | |
| 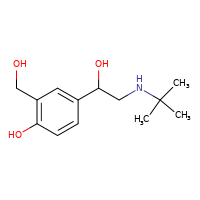  Salbutamol | a)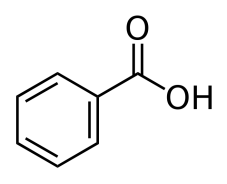Benzoic acid^2,4^ | | 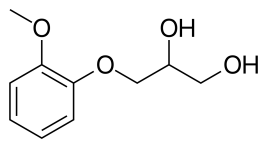  b) 3-(4-methoxyphenoxy)-1,2-propanediol^6^ | | | |   c) Ethyl 2-(4-nitrophenoxy)benzoate^3^ | |
| 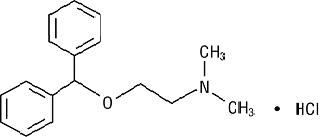  Diphenyl Hydramine in Uphadyl Forte | 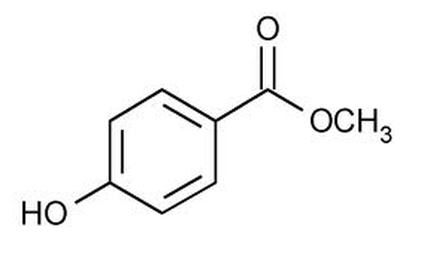  a) Methylparaben^2,3^ | | |   b) 5-methyl-2(3H)-furanone^1^ | | | | |
| 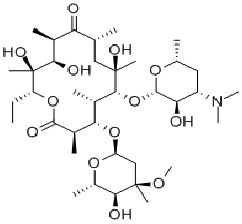  Erythromycin | 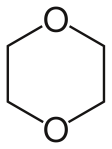  a) 1,4-Dioxane^4,6^ | |   b) Methyl butanedioc acid ester^2^ | | |   c) 3-(1-Methoxy-1-methylethoxy)-2-methylpropionic acid^1,3^ | | |
| 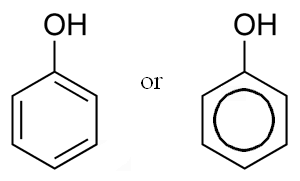 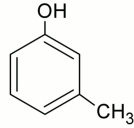  Insuman Rapid |   a) 2,4,6-trichlorophenyl tridecyl fumaric acid ester^1^ | | |   b) 13-(E)-Docosenoic acid^3^ | | | | |
| 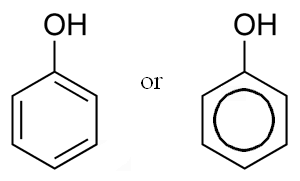 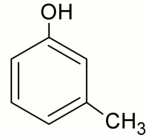  Insuman Basal |   a) Benzoic acid, 4-methyl-2-trimethylsilyloxy ester^3^ | | | | | | | |
| 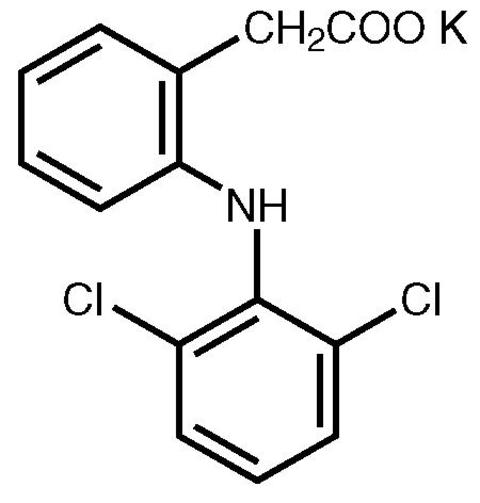  Cataflam/ Diclofenac |   a) 2-ethylbutyl octyl-2,6-pyridinedicarboxylic acid ester^1^ | | | | 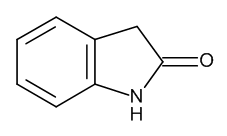  a) 1,3-dihydro-2H-Indol-2-one^2,3^ | | | |
| 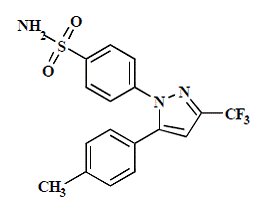  Celebrex | 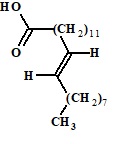  a) 13-(Z)-Docosenamide^1^ | | | |   b) 5-Methylbenzimidazolo[2,1-a]phthalazine^2,3^ | | | |
| 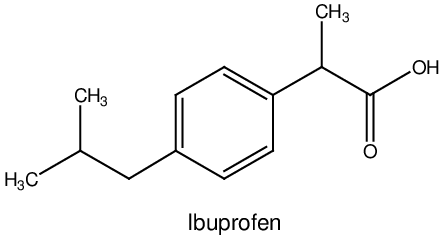  Ibuprofen |   a) 6-amino-2-(4-methylphenyl)- naphthalimide^1^ |   b) 1-(p-fluorophenyl)-anthraquinone^2,3^ | | | c) N-propxycarbonyl-butyl-L-Valine ester^4^ | | | 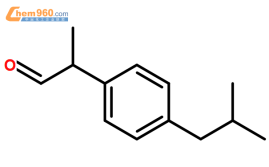  d) α-methyl-4-(2-methylpropyl)-benzeneacetaldehyde^5^ |
| 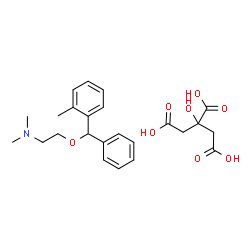  Orphenadol | 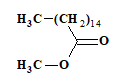  a) Methyl palmitate^2,3^ | | | | | | | |
| 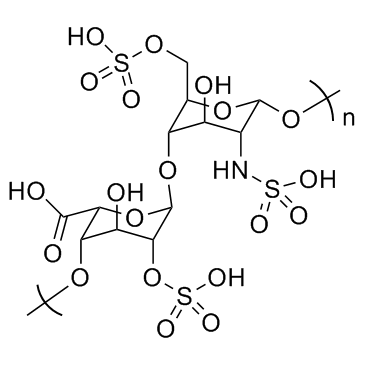  Heparinol | 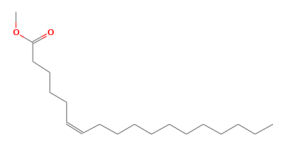  a) 6-Octadecenoic acid^1^ | | | | 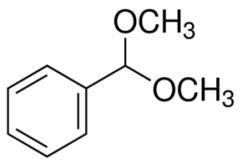  b) Dimethyl acetal benzaldehyde^2,3^ | | | |
| 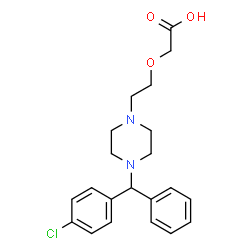  Cetirizine | 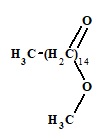  a) Methyl palmitate^1,2,3^ | | | | 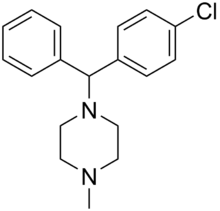  b) Chlorcyclizine^1,2,3^ | | | |

1: Laccase; 2: Laccase and H_2_O_2_; 3: H_2_O_2_; 4: Apo-Tp; 5: TpCuS 12; 6: NpCuC 12 catalysts

**S3 Table. Raw data obtained from GCMS spectra for the mentioned PhACs and catalysts**

| PhACs  Catalysts | Laccase | | Laccase + H_2_O_2_ | | H_2_O_2_ | | Tp | | Tp-CuS 12 | | Np-CuC 12 | |
| --- | --- | --- | --- | --- | --- | --- | --- | --- | --- | --- | --- | --- |
|  | R_T_/  min | Area x 10^6^* | R_T_/  min | Area x 10^6^* | R_T_/min | Area x 10^6^* | R_T_/min | Area x 10^6^* | R_T_/min | Area x 10^6^* | R_T_/min | Area x 10^6^* |
| o-cresol | 12.1 | 0.95/  [39.70] | 18.5 | 1.67/  [46.89] | 5.5 | 14.59/  [812.84] | No oxidation | | 2.5  3.9  5.5 | 0.15/  0.51/  0.06/  [0.85] | No oxidation | |
| m-cresol | No oxidation | | | | | |  |  | 3.9  11.8 | 0.52/  0.89/  [549.22] |  |  |
| p-cresol |  |  |  |  |  |  |  |  | 3.9 | 0.55/  [585.38] | 15.6  16.8  17.2 | 8.63/  25.20/  4.89/  [1531.92] |
| 2,4-dichlorophenol |  |  |  |  |  |  |  |  | 3.9 | 0.44/  [7.52] | No oxidation | |
| Salbutamol | 6.1  6.4 | 6.51/  80.70/  [87.20] | 7.9 | 2.86/  [3.36] | 6.1  6.4 | 1.12/  23.46/  [24.57] | 7.8 | 38.82/  [40.09] | 3.9 | 0.06/  [0.12] | 14.6-16.1 | 43.26/  [46.80] |
| Uphadyl Forte | 7.8 | 30.10/  [309.98] | 11.9 | 7.81/  [14.36] | 7.8  9.2  11.7 | 21.29/  25.13/  26.32/  [298.85] | No oxidation | | | | | |
| Erythromycin | 9.7  19.4 | 4.01/  2.76/  [8.99] | 8.9  9.7 | 5.37/  7.67/  [38.35] | 9.7 | 3.28/  [72.71] | 8.4  8.9 | 5.79/  4.18/  [17.41] | 13.3  14.2  14.4 | 2.02/  0.81/  9.13/  [25.10] | 8.7  9.2 | 10.11/  7.79/  [51.51] |
| Insuman Rapid | 21.9 | 2.96/  [4.55] | 9.7 | 8.76/  [21.99] | 9.6 | 24.42/  [29.25] | No oxidation | | | | | |
| Insuman Basal | 24.0 | 0.41/  [0.41] | 9.7 | 4.52/  [9.30] | 9.7 | 3.62/  [14.28] |  |  |  |  |  |  |
| Diclofenac/ Cataflam | 21.9 | 0.59/  [6.55] | 19.3 | 22.69/  [23.27] | 19.3 | 40.36/  [49.19] |  |  |  |  |  |  |
| Celebrex | 23.9 | 5.26/  [419.71] | 17.1 | 9.62/  [17.16] | 17.1 | 0.31/  [0.47] |  |  |  |  |  |  |
| Ibuprofen | 17.1 | 0.29/  [0.29] | 17.1 | 0.15/  [0.15] | 17.1 | 0.27/  [0.27] | 24.6 | 67.77/  [226.88] | 18.3 | 0.36/  [1.02] | 13.1  13.7 | 3.81/  2.83/  [185.78] |
| Orphenadol | 24.3 | 0.02/  [0.15] | 16.8 | 2.20/  [610.75] | 16.8 | 2.03/  [471.73] | No oxidation | | | | | |
| Heparinol | 5.8  21.9 | 12.10/  1.86/  [131.23] | 5.8  6.9 | 106.92/  2.78/  [115.92] | 5.8  6.9 | 142.70/  4.72/  [153.70] |  |  |  |  |  |  |
| Cetrizine | 11.8  20.2 | 24.28/  0.91/  [53.19] | 11.8  20.2 | 26.36/  1.04/  [83.12] | 11.8  20.2 | 26.90/  1.29/  [114.06] |  |  |  |  |  |  |

**S1 Scheme. Solid phase peptide synthesis of Tp**

(a)


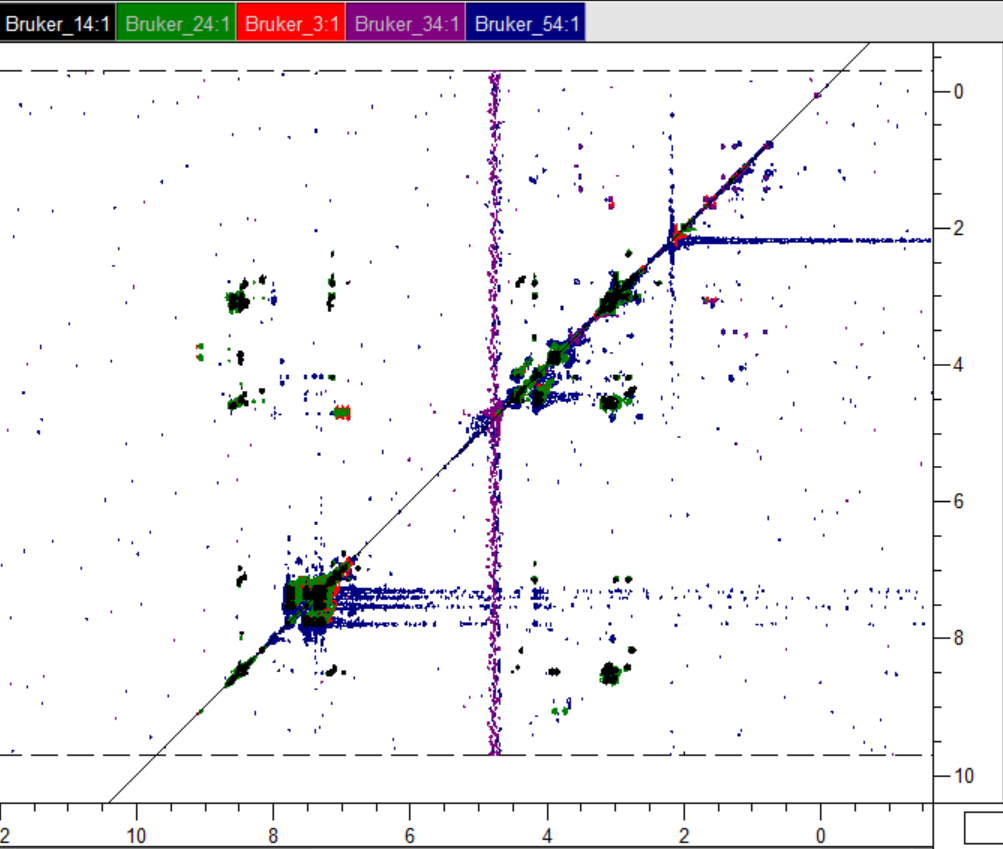


His aromatic Hδ & Hε

-NH-, H_α_

H_α_, -NH-

H_α_

H_β_

**
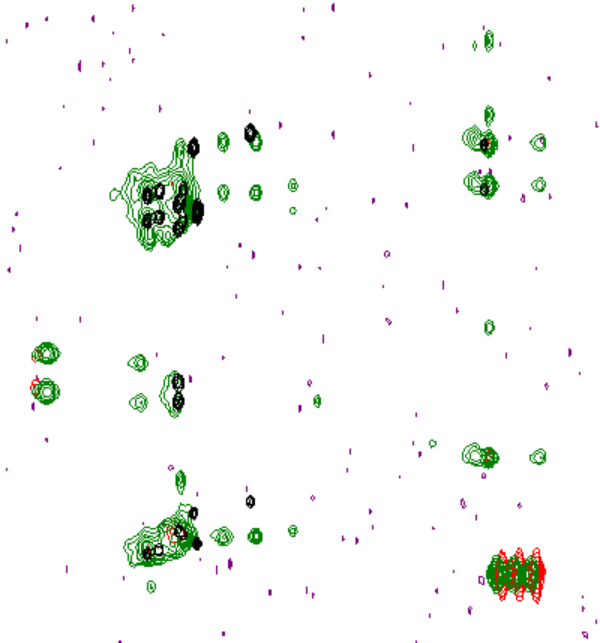

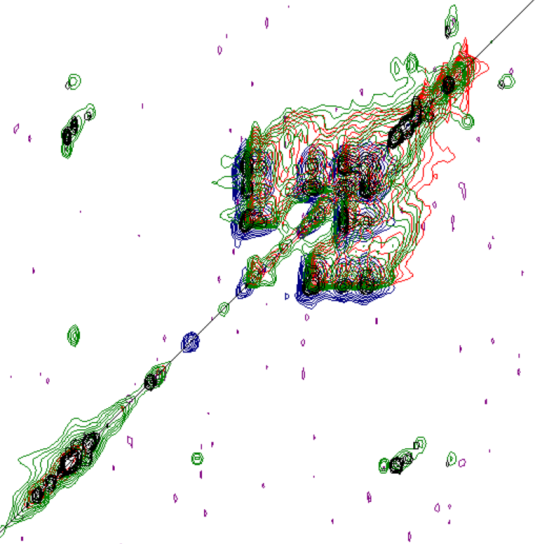

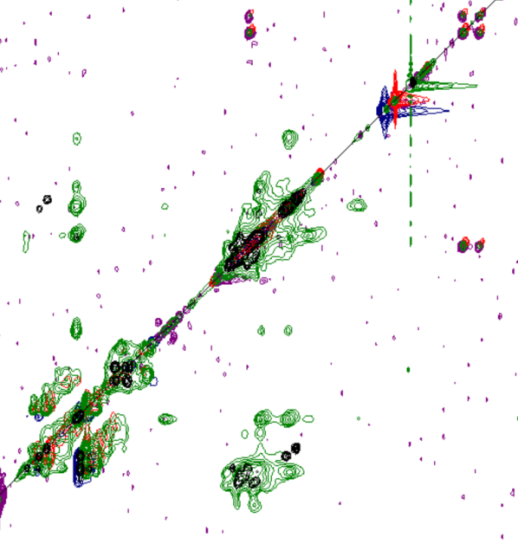
**

**Black:** Np, **Green:** NpCuS 41, **Red:** NpCuS 14, **Purple:** NpCuC 14, **Blue:** NpCuC 41

TOCSY spectra at D9=80ms.


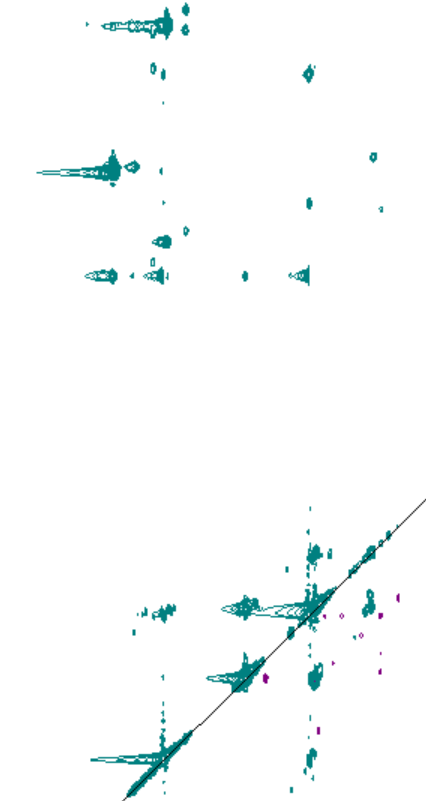

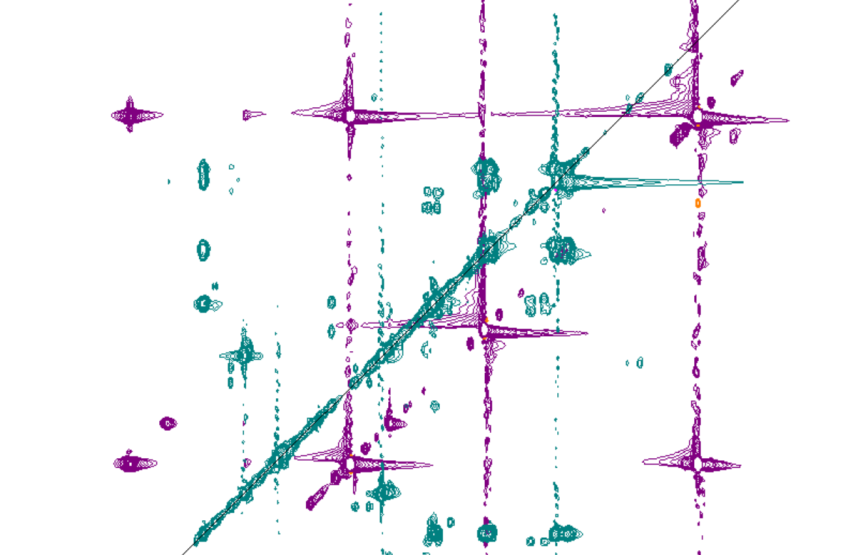


H_β_, H_α_

(b)


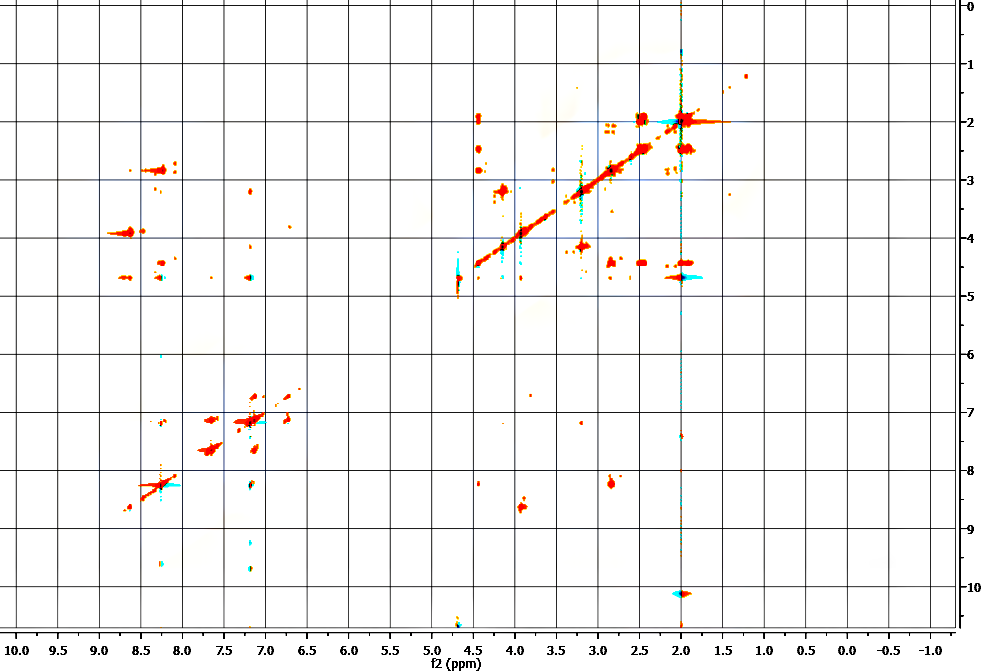


-NH-, H_α_

Aromatic His protons

H_α_, H_β_

H_β_, H_β_, -SH-

H_β_, H_α_

**Cyan green**: Tp; **Orange red**: Tp-CuS 1:2

(c)

**S1 Fig. ^2^D NMR TOCSY and HSQC of peptides overlapped with their Cu-peptides** **at pH 6 and 4°C.**

**(a) ^1^H-^1^H TOCSY of Np, Np-Cu(CH_3_COO)_2_ and Np-CUSO_4_ in 4:1 and 1:4 molar ratio.**

**(b) ^1^H-^1^H TOCSY of HMGC (Tp) and Tp-CuSO_4_ (1:2)**

**(c) ^13^C-^1^H HSQC of Np**
